# Student Life in the Sixteenth Century

**Published:** 1894-09-22

**Authors:** 


### Student Life in the Sixteenth Century.

About 1540 Professor J. Sylvius, Regius Professor of Medicine at the University of Paris, and a well-known anatomist, being troubled in his kindly soul at the privations and consequent ill-health which some of his students endured, published a book entitled "De Victu facile Pauperum Scholasticorum"—"An Easy Way of Life for Poor Scholars." He guides the poor scholar from dawn to dusk, or even later, as Rabelais tells us the canonical hours for a student to keep in those days were, "rise at five, take dinner at nine, supper at five, and go to bed at nine." No mention is made of breakfast, nor of any lunch to break the eight hours' fast between dinner and supper. But meals were heavier and heartier in the middle ages than now. This must have been, for Professor Sylvius, beginning his advice to the poor scholar at the moment of his awakening, bids him consider, before he gets up, the quantity and quality of the supper he ate at five o'clock the evening before, which, it seems, may possibly remain undigested. In this case the student is advised to remain in bed, and keep his hands and arms upon his stomach, "as this favours digestion." That he may not, however, waste his time by so doing, the Professor gives him advice as to reading in bed. "If it is cold cover the upper part of your body well, and sit up with the rest covered by the bed clothes, and so read for a time, the taper being protected by a horn lantern."

It is likely enough that cold, as well as indigestion, made many students do their work in bed. Sylvius advises them to dispense with a fire, except in the severest weather, as being harmful to the body, and the fire he recommends would need cold weather indeed to make it attractive. It is to be built in the middle of the chamber, and elevated in an iron pot, that our shivering student may get full benefit of the heat. Coal—*carbo terræ*—is admitted to be the best fuel, but it is discarded on account of its smoke, which ruins clothes and pollutes food cooked above it. If employed, Sylvius insists that an *emissarium* should be provided for the escape of the smoke, and he regards wood as a preferential fuel. He particularly condemns the extravagant and new-fangled practice of building the fire by the wall or on the hearth, whereby much precious warmth must needs be wasted.

The heat given by the bed-clothes must have been accompanied by fewer inconveniences than that thrown out by such a fire, but the bed-clothes themselves seem to have been very generally insufficient. Thus Professor Sylvius recommends that on going to bed the student should, to avoid cold feet, wrap them in the warmest part of his breeches, and he adds another suggestion: "If you fear cold at night, tie the lower corners of the bed together with a cord; the vulgar call it 'making a helmet.'" He bids his reader use thick wraps for blankets, "and for an outer covering a hempen sack filled with chaff, straw, or hay will be of great use." No degrading luxury here!

From his bed the Professor accompanies the student through all the details of his toilet. On getting up he is to stand on the warmest part of his bed, and there rub himself vigorously to restore the circulation. Having donned his clothes he is to comb his hair with a clean comb, aiding its action with his fingers. While doing so he is to walk about, and this is considered a suitable opportunity for saying his prayers. Some sensible advice is also given as to attending to the functions of the body, lest constipation and headache should result from neglect. But we note one curious omission in these toilet counsels; nothing is said about washing. Not that Professor Sylvius was a dirty man. On the contrary, he distinctly says that you cannot keep your feet warm unless you keep them clean, and therefore advises their being washed "often—about once a month!" He also recommends the rinsing of mouth and hands after dinner in either water or water mixed with wine. And that is the beginning and end of his ablutions.

With regard to food the student is expected to be largely a vegetarian. "If your circumstances allow you to eat meat," says Sylvius, as if that were rather unlikely, "choose pork, for that is cheapest, but take little, and not of an old pig, nor long salted." For animal food fish—carp, &c.—is mentioned as cheap but insipid, a fault, however, which a slight smoking will cure. But fruit and cereals are to be the student's mainstay. Wholesome, cheap, and readily made dishes may be got thus: "Take wheaten or half-wheaten bread, well kneaded, moderately fomented, and not salt. Make it into a gruel with hot water. If you can get some fat, such as lard, or purified pig's fat, or even butter to flavour it"—why *even* butter, O Professor?—"it will be the more palatable. I do not advise you to use flour in place of bread, but you may cook flour, barley, oatmeal, rice, or millet with a little oil or butter, aniseed or leeks. Cow's milk, alone or with water, is wholesome cooked with bread or barley grains. Some make a porridge of cheese; but all cheese tends to produce stone"—here's news for the pathologists!—"so abstain from it, though highly nutritious, unless you are accustomed thereto, and so it has become harmless. If you like a simple broth, an onion cooked in water with a little salt will quiet a barking stomach; or, if you think that too heating, try spinach, lettuce, purslain, cucumbers, gourds, and the like. Apples and pears, cooked with water and bread, with a little butter and salt, will make a wholesome and pleasant food, though if taken in excess, especially in winter, they produce crude humours." Fresh eggs, soft boiled, are recommended, and "chestnuts, roast or boiled, almonds, filberts, raisins, plums, and dried figs are useful, especially on fast days."

For drink plain water is nowhere recommended, even to the poor student, and recalling what Seine water is at the present day it was probably wise not to venture on it in the insanitary mediæval times. Failing wine, which may be too expensive, the Professor recommends beer, or the liquid in which prunes, raisins, liquorice, or barley have been cooked. Honey wine, made according to Pliny's recipe, is also mentioned favourably. "For dessert, if you can live so luxuriously, plums, grapes, raisins, cooked apples and pears, alone or with butter and fennel, are best." The fennel seems to modern taste unnecessary, but perhaps was meant to add a solidity, of flavour at least, to a dietary which seems somewhat too slender for men who are attacking the severest studies, and are forced to learn them in a dead language.

The most valuable part of Professor Sylvius' treatise is his advice concerning the hearing of lectures. He demands complete attention from the student, but observation rather than note-taking. "Observe attentively the lecturer's method and order of discourse, and the reason for it, and write the headings in a few notes, which you may afterwards go over alone, or with a friend, and commit to memory. . . . Some follow every word with the pen, but since their minds are wholly given to their writing, they are apt to notice little what is said, unless they go over it carefully afterwards." This advice is as true and just to-day as it was three hundred and fifty years ago. And perhaps the counsel as to eating and sleeping may be useful to some poor students even now. But we hope that once at least in every session Professor Sylvius invited his students to such a supper as they would remember, and with satisfaction, even twelve hours afterwards, or more.

